# Occurrence of aflatoxins and its management in diverse cropping systems of central Tanzania

**DOI:** 10.1007/s12550-017-0286-x

**Published:** 2017-08-07

**Authors:** Anitha Seetha, Wills Munthali, Harry W. Msere, Elirehema Swai, Yasinta Muzanila, Ethel Sichone, Takuji W. Tsusaka, Abhishek Rathore, Patrick Okori

**Affiliations:** 1 International Crops Research Institute for the Semi-Arid Tropics (ICRISAT), P.O. Box 1096, Lilongwe, Malawi; 2 Agricultural Research Institute, Hombolo (ARI-Hombolo), Central Zone Crop Research, P.O.BOX 299 Dodoma, Tanzania; 3 Sokoine University of Agriculture (SUA), P.O Box 3000, Chuo Kikuu, Morogoro, Tanzania; 4 International Crops Research Institute for the Semi-Arid Tropics (ICRISAT), Patancheru, Telangana 502 324 India

**Keywords:** Post-harvest management, Aflatoxin contamination, Crop diversity, Food safety, Sub-Saharan Africa, Tanzania, Confounding factor

## Abstract

The staple crops, maize, sorghum, bambara nut, groundnut, and sunflower common in semi-arid agro-pastoral farming systems of central Tanzania are prone to aflatoxin contamination. Consumption of such crop produce, contaminated with high levels of aflatoxin B_1_ (AFB_1_), affects growth and health. In this paper, aflatoxin contamination in freshly harvested and stored crop produce from central Tanzania was examined, including the efficacy of aflatoxin mitigation technologies on grain/kernal quality. A total of 312 farmers were recruited, trained on aflatoxin mitigation technologies, and allowed to deploy the technologies for 2 years. After 2 years, 188 of the 312 farmers were tracked to determine whether they had adopted and complied with the mitigation practices. Aflatoxigenic *Aspergillus flavus* and aflatoxin B1 contamination in freshly harvested and stored grains/kernels were assessed. *A. flavus* frequency and aflatoxin production by fungi were assayed by examining culture characteristics and thin-layer chromatography respectively. AFB_1_ was assayed by enzyme-linked immunosorbent assay. The average aflatoxin contamination in freshly harvested samples was 18.8 μg/kg, which is above the acceptable standard of 10 μg/kg. Contamination increased during storage to an average of 57.2 μg/kg, indicating a high exposure risk. Grains and oilseeds from maize, sorghum, and sunflower produced in aboveground reproductive structures had relatively low aflatoxin contamination compared to those produced in geocarpic structures of groundnut and bambara nut. Farmers who adopted recommended post-harvest management practices had considerably lower aflatoxin contamination in their stored kernels/grains. Furthermore, the effects of these factors were quantified by multivariate statistical analyses. Training and behavioral changes by farmers in their post-harvest practice minimize aflatoxin contamination and improve food safety. Moreover, if non-trained farmers receive mitigation training, aflatoxin concentration is predicted to decrease by 28.9 μg/kg on average.

## Introduction

In semi-arid agro-ecologies of central Tanzania, most farmers practice inter-cropping or mixed cropping systems that involve diverse drought-tolerant crops such as bambara nut (*Vigna subterranea* (L.) Verdc.), sorghum (*Sorghum bicolor* (L.) Moench), sunflower (*Helianthus annus* L.), maize (*Zea mays*, L.), cowpea (*Vigna unguiculata* (L.) Walp), pigeonpea (*Cajanus cajan* L. Millsp.), and groundnut (*Arachis hypogaea* L.). These crops are susceptible to infection by *Aspergillus* spp., which are fungi that produce a group of toxins known as aflatoxins (Guchi 2015). Specifically, *A*. *flavus* is the major aflatoxin B_1_ (AFB_1_) producing species, which predominately contaminates oilseeds, cereals, grain legumes, and tree nuts (Klich 2007). The warm and humid climate common to the tropical semi-arid agro-ecologies of sub-Saharan Africa are particularly conducive to infection of crop produce by *Aspergillus* spp. and subsequent contamination with aflatoxins (Bosch et al. 2004; Yu and Yuan 2004).

Chronic exposure to low or moderate amounts of AFB_1_ through consumption of contaminated food products can cause liver cancer (Wild 2007), immune suppression (Jolly et al. 2013), and stunted growth in children, as reported previously (Gong et al. 2003; Williams et al. 2004). Acute exposure through consumption of highly contaminated crop produce can cause sudden death (Williams et al. 2004; Wagacha and Muthomi 2008; Klich 2007; WHO 2006; Hall and Wild 1994). The central region of Tanzania has one of the highest stunting levels in the country, with Dodoma district reporting over 40% stunting in children under the age of 5 years (TFNC 2014). A recent study conducted in Iringa, Tabora, and the Kilimanjaro regions of Tanzania, showed that 67% of children had serum aflatoxin biomarkers with a mean aflatoxin-albumin adduct concentration of 4.7 pg/mg of albumin. However, the causal relationship between the presence of aflatoxin-albumin biomarkers in the blood and stunting in children has not been established in these regions (Shirima et al. 2015). In addition to its effects on health, AFB1 contamination in crop produce reduces opportunity to access lucrative export markets where strict AFB1 contamination levels are prohibitive, moderated, and monitored (Otsuki et al. 2001). The economies of many sub-Saharan African countries, being largely agrarian, with limited management of AFB1 contamination, thus, miss the opportunity to engage in competitive markets (Monyo et al. 2012).

Few studies have examined AFB1 contamination and the impacts of its mitigation on commonly cultivated crops from complex cropping systems such as those in Tanzania (Kimanya et al. 2008) and many regions of sub-Saharan Africa. A recent study conducted in Tanzania reported that 18% of maize produce was contaminated with aflatoxins, with levels of up to 158 μg/kg, and 12% of the samples had over 10 μg/kg of AFB1, the Tanzania maximum allowed limit (Kimanya et al. 2008). The scope of crops studied previously is limited, and no studies have evaluated bambara nut, sunflower, and other crops associated with semi-arid cropping systems of Tanzania.

The crops examined in this study thus provide a framework for investigating the role of crop production environment and storage micro-environments on AFB_1_ contamination. Post-harvest crop handling of the crops included in this study, from the field to homestead inadvertently increase risk of *Aspergillus* colonization of grain and kernels and its related AFB_1_ contamination (Tsusaka et al. 2016). Crop diversity may also influence *Aspergillus* population dynamics, affecting the ratio of aflatoxigenic to non-aflatoxigenic strains and *Aspergillus* spp. and therefore the level of contamination (Mehl and Cotty 2013).

Previous studies identified factors causing aflatoxin contamination in various crops (Hell et al. 2000; Turner et al. 2005; Wu and Khlangwiseta 2010). To the authors’ knowledge, however, the effects of such factors on contamination have not been quantified in sub-Saharan Africa. Availability of reliable information regarding AFB_1_ contamination in crops harvested from complex cropping systems is essential to develop effective mitigation programs. Accordingly, the objectives of this study were to (1) determine the frequency of AFB_1_ contamination in bambara nut, sunflower, sorghum, maize, and groundnut in both fresh and stored crop produce and (2) quantitatively investigate the outcomes of the training on adoption of post-harvest crop management technologies by farmers on AFB_1_ contamination.

## Materials and methods

### Characterization of cropping and storage systems

The study was conducted in five villages in central Tanzania: Njoro in the Manyara region (Kiteto district), and Chitego, Mlali, Moleti, and Laikala in the Dodoma region (Kongwa district). A baseline assessment was conducted in each village during which the primary data was gathered on the cropping systems and livestock production systems, while the secondary data on the agricultural sector, demography, and market information from each district was used to characterize existing farming systems. A total of 312 farmers were recruited in 2013 and the study conducted in 2014 and 2015. The farms were geo-referenced using a geographical positioning system. Rain gauges were installed in each village to monitor rainfall distribution. Typically, this region receives approximately 300 mm rainfall in unfavorable years and 500 mm in favorable years.

### Collection of soil samples to investigate *Aspergillus* species diversity

To identify sources of AFB_1_ contamination in the field, 312 composite soil samples were collected from each farmer and assayed for *Aspergillus* spp. in May 2013. Each composite soil sample was generated by mixing soil sub-samples collected from 50 m^2^ in each farmers’ field. During sampling, each sample area was divided into quarters and samples collected along a diagonal in each quarter. Soil sampling was done along a gradient to capture spore and other fungal propagules dispersal by storm rain water. At each sample station, approximately 5 g of soil was collected from a depth of 2–10 cm. A total of 12 samples per field were subsequently bulked and quartered to generate a composite sample per farmer (Jaime-Gracia and Cotty 2006). These samples were secured in paper bags and stored at 5 °C until further processing.

### Isolation and characterization of *A. flavus*

Two assays involving colony pre-screening to eliminate non-*Aspergillus* spp. followed by assays for AFB_1_ production to confirm aflatoxigenicity were conducted to characterize *A. flavus* isolates (Abbas et al. 2004). The soil samples were air-dried and ground into a fine powder using a pestle and mortar. Ten grams of each soil sample was divided into 3 g portions, generating 3 replicates per sample. Each replicate sample was added to modified Dichloran Rose Bengal Chloramphenicol medium (Sigma-Aldrich, St. Louis, MO, USA) in Petri dishes and incubated for 4–7 days at 25 °C (Horn et al. 1995). *Aspergillus* species were identified according to Klich and Pitt (1988), and their population densities quantified on soil dry weight basis.

Subsequently, *A*. *flavus* was purified from original culture plates and plated on coconut-agar medium to investigate aflatoxin production (Lin and Dianese 1976). Fresh coconut extract was prepared by grinding a 2:1 mixture of distilled water and fresh coconut-flesh in a blender; boiling to skim off the oil, and then filtering through cheesecloth to obtain the fresh filtrate for augmenting the agar (BD Biosciences, Franklin Lakes, NJ, USA). The culture plates were incubated for 4–7 days at 30 °C without light. After 5 days, samples were assayed for presence of AFB_1_ using UV light (Sudini et al. 2015) and AFB_1_ production confirmed by thin-layer chromatography (TLC) (Park et al. 1994; Abbas et al. 2004).

### Collection of crop produce to study aflatoxin contamination

Representative samples of crop produce were collected at two different times. First, samples were collected from standing crops in fields just before harvest in May/June of 2013 and 2014. Samples were collected along a 50 m transect using an approach similar to that used for soil sample collection. At each sample station, 10–12 cobs/ears of each crop were collected. Depending on field size, samples were pooled and quartered to generate one composite sample of 1 kg, air-dried to a constant weight to reduce moisture, placed in paper bags, and stored at 5 °C until further analysis (Mahuku et al. 2010). Farmers who provided field crop samples also provided stored samples of their crop produce for the study. These stored samples were those from the same fields in which samples of freshly harvested crop produce had been collected previously and stored for at least 5 months, the typical storage duration in the study area. During sample collection, a representative sample was obtained by mixing 10 samples, each weighing approximately 10 g, collected from different parts of each storage bag to constitute 100 g of sample and was used to evaluate AFB_1_ contamination.

### Determination of AFB1 from grains/kernels

The 100 g sub-samples were weighed ground into a fine powder, and two replicate samples of approximately 20 g of each sample were mixed with 100 mL of 70% methanol/distilled water (*v*/*v*) containing 0.5% potassium chloride. The mixture was transferred to a 250-mL conical flask, shaken at 300 rpm for 30 min (Gallenkamp Orbital Shaker, CAT # SCM 300 0101, Weiss Technik, Grand Rapids, MI, USA) and filtered through Whatman No. 41 filter paper (GE Healthcare, Little Chalfont, UK). The filtrate was assayed for AFB_1_ using an in-house indirect competitive enzyme-linked immunosorbent assay (ELISA) (F96 MaxiSorp, Thermo Fisher Scientific, Waltham, MA, USA) at a detection limit of 1 μg/kg and mean recovery of 92.5% (Reddy et al. 2001). The method was validated with naturally contaminated corn reference materials (4.2 and 23.0 μg/kg AFB_1_, product no. TR-A100, batch no A-C-268 and A-C 271; R-Biopharm AG, Darmstadt, Germany). This method has high reproducibility with mean percentage recovery of 92.5% of AFB_1_ (Reddy et al. 2001). Briefly, the samples were tested using a polyclonal antibody produced against AFB_1_-BSA. Alkaline phosphatase-conjugated anti-rabbit antibodies (Sigma-Aldrich) were used as the secondary antibodies, and *para*-nitrophenyl phosphate (Sigma-Aldrich) was used as a substrate. Colorimetric reaction was measured using an ELISA plate reader (Multiskan reader, Thermo Fisher Scientific) using a 405-nm filter. To further confirm the presence of AFB_1_ in selected samples, the filtrate was subjected to thin-layer chromatography using silica gel-coated 20 × 20 cm glass plates (Fluka Analytical, Sigma-Aldrich), developed in chloroform: acetone (93:7, *v*/*v*) under vapor saturated conditions, and detected directly under long-wave UV light based on fluorescence (Park et al. 1994; Abbas et al. 2004).

### Tracking farmer learning and adoption of aflatoxin mitigation technologies

Following the collection of crop and soil samples, intervention activities for mitigation, awareness, and technology promotion were undertaken for 2 years (2013–2014). The mitigation technologies included (1) ventilated drying of groundnuts in the field (the Mandela cork method (ICRISAT 2012), (2) ventilated drying of other grains/kernels on polythene sheets to avoid exposure to soil, (3) hand-sorting of grain/kernels before processing into various food products, (4) minimization of wet shelling, during which pods were soaked for 5–10 min for softening and ease shelling by hand, and (5) ventilated storage of well-dried groundnuts and other kernels/grains in moisture-free, dry wooden pallets. This ventilated drying system allows air to flow through stacked haulms with pods, slowly drying the nuts and preventing the spread of the *A*. *flavus*. Focus group discussion, field demonstrations, and farmer learning sessions were organized through the learning-by-doing approach for technology promotion and knowledge dissemination. In June 2015, 188 of the 312 farmers involved in the study were tracked to assess the outcome of farmer learning and adoption of correct post-harvest management practices in grain/kernel handling compliance of AFB_1_ mitigation practices. Crop samples were obtained to assess the frequency of AFB_1_ contamination in stored samples to compare with the baseline.

### Data analysis

Data from the baseline and farmer learning sessions were coded and subjected to statistical analysis using SPSS version 16 (SPSS, Inc., Chicago, IL, USA), R version 3.1.1 (R Development Core Team 2014), SAS version 9.4 (SAS Institute, Inc., Cary, NC, USA 2013), and STATA version 14. AFB_1_ levels were tested to determine the statistical significance of differences between various samples using the two-sample *t* test by considering that samples showed independent assuming unequal variance (Cressie and Whitford 1986). Furthermore, multiple linear regression was performed to identify the effect of each factor associated with AFB_1_ contamination by controlling for other covariate variables (Dismuke and Lindrooth 2006), enabling differentiation between actual factors and spurious associations. Four dummy variables coded as 1 if the sample was (1) from geocarpic structures, i.e., from (“groundnut-bambara nut dummy”), (2) an oilseed crop, (3) a stored crop sample, and (4) taken after training of farmers, but otherwise coded as 0, were generated and included in the ordinary least squares regression with a sample size of 2485.

## Results

### Characteristics of cropping and storage management systems

Most households (95%) practiced mixed cropping, with maize as the major staple crop. Farmers also grew sunflower, sorghum, groundnut, and bambara nut for food and income on sandy loams in Moleti, Mlali, and Laikala and clay loams in Chitego and Njoro. These crops are produced either as a sole crop, particularly on large farms with ≥2 ha of land, or as mixed crops on farms with ≤2 ha of land holding. Baseline analysis revealed that in general, farmers dried their crops produce on rooftops or on bare earth at home. Sunflower, because of its bulkiness, was exclusively dried on the ground, in most cases, directly on soil surface. Only 34% of recruited farmers adequately dried their harvested crop produce, and only 36% the farmers used recommended storage technologies. At least 79% of farmers graded their crops based on grain or pod size rather than using the health and quality of grains and pods. Only 14% of farmers discarded rotten or damaged produce while others either consumed or fed this produce to their livestock (Table 1).

**Table 1:** Changes in farmer knowledge, attitude, and practice (KAP) awareness on AFB_1_ and the needed mitigation practice before and after training

KAP items captured during survey	Before training proportion (%) *n* = 312	After training proportion (%) *n* = 188
Awareness of AFB_1_	30.0	82.0
Farmers who grade the grains before storage	23.0	84.0
Farmers who remove rotten grains before storage	14.0	78.0
Awareness of methods of drying, grading and storage	*14.0*	92.0
Farmers drying grains just because it is traditional practice	100.0	0.0
Farmers who practice proper drying methods	34.0	82.0
Farmers who practice proper storage methods	36.0	82.0
Farmers who throw away the grade out	15.0	35.
Farmers who utilize the grade outs in alternate ways	75.	42.
Farmers who feed the grade outs to livestock	10.	23.

### Occurrence of *A*. *flavus* in farm soil

**Fig. 1:**
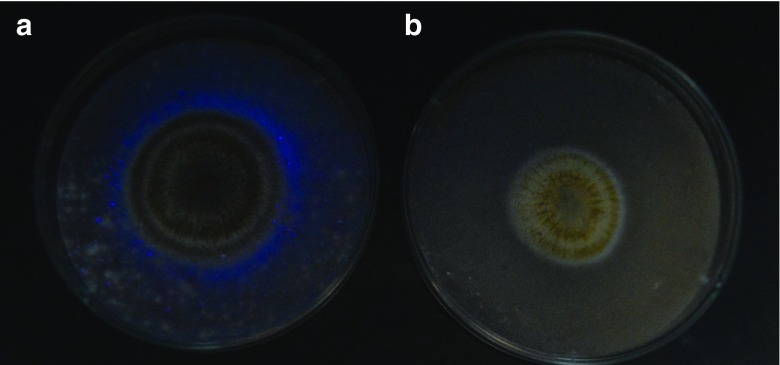
Presence of (**a**) aflatoxigenic *A. flavus* showing *blue* fluorescence surrounding the colonies under UV light and (**b**) negative control of *A. flavus* colonies that do not produce fluorescence under UV light

Each of the 312 soil samples tested contained *A*. *flavus.* The population density of *A*. *flavus* per 1 g of soil ranged from 3.4 log colony-forming units in Moleti and Njoro to 4.1 log colony-forming units in Laikala. The presence of aflatoxigenic *A*. *flavus* was identified by the presence of blue florescence surrounding fungal colonies under UV light (Fig. 1). Prescreened cultures were confirmed for AFB_1_ production by TLC. The frequency of *A*. *flavus* was the highest in samples from Chitego at 84% (45/53 samples assayed), followed by those from Njoro at 71% (35/49 samples assayed) (Table 2).

**Table 2:** Levels of AFB_1_ contamination in grain/kernels of selected crops from a semi-arid agroecology of central of Tanzania based on AFB_1_ levels in fresh sample material and AFB_1_ levels in stored sample material and frequency of aflatoxigenic *A*. *flavus*

Village	Frequency of aflatoxigenic *A*. *flavus* (*n*/*N*)	Crops	AFB_1_ in fresh sample		AFB_1_ in stored sample		*t* test
			Mean ± SE	Maximum	Mean ± SE	Maximum	*t* statistic
Chitego	84 (45/53)	Bambara nut	1.5 ± 0.69	10.7	38.07 ± 5.94	74.8	6.11**
		Groundnut	12.0 ± 20.7	62.0	21.9 ± 14.4	56.1	4.7**
		Maize	na	na	na	na	na
		Sorghum	7.6 ± 6.4	23.4	9.1 ± 6.7	62.5	0.15 ns
		Sunflower	4.8 ± 1.5	43.0	19.0 ± 12.2	605	1.15 ns
Laikala	36 (10/28)	Bambara nut	1.3 ± 0.46	13.7	3.96 ± 1.0	14.3	2.72**
		Groundnut	32.0 ± 66.4	278.0	84.9 ± 114.4	427.0	2.7*
		Maize	0.09 ± 0.1	1.2	0.76 ± 0.17	2.4	3.64**
		Sorghum	0.35 ± 0.2	10.7	2.7 ± 1.3	29.8	1.7 ns
		Sunflower	1.76 ± 1.7	63.0	61.1	489.3	1.5 ns
Mlali	50 (22/44)	Bambara nut	35 ± 114.0	411.4	207.3 ± 206.0	567.8	2.28*
		Groundnut	21.8 ± 14.1	84.8	85.4 ± 99.0	298.2	5.2^**^
		Maize	0.03 ± 0.01	12.2	2.8 ± 1.2	21.9	2.17*
		Sorghum	1.00 ± 0.3	10.0	25.7 ± 17.3	70.0	1.43*
		Sunflower	1.7 ± 0.6	26.0	4.9 ± 1.7	43.7	1.76*
Moleti	50 (10/20)	Bambara nut	0.7 ± 1.3	75.0	29.2 ± 24.7	105.0	6.1**
		Groundnut	48.2 ± 41.06	868.2	377.3 ± 163.7	3297.3	1.95*
		Maize	0.9 ± 2.3	2.3	4.2 ± 9.5	43	2.76*
		Sorghum	0.9 ± 0.5	2.0	9.4 ± 3.5	73.9	2.3*
		Sunflower	1.0 ± 0.3	2.7	99.9 ± 20.6	425.4	4.8**
Njoro	71 (35/49)	Bambara nut	1.7 ± 1.4	4.4	41.6 ± 22.6	215.5	2.4*
		Groundnut	15.6 ± 6.3	145.4	289.7 ± 75.0	1178.8	3.64**
		Maize	1.1 ± 0.5	23.8	2.5 ± 0.5	29.2	1.71*
		Sorghum	3.5 ± 0.45	10.0	93.3 ± 12.3	138.7	3.7*
		Sunflower	6.9 ± 5.9	294.8	82.0 ± 21.3	294.8	3.95**

AFB1 contamination was estimated using ELISA (Monyo et al. 2012), which has a lower detection limit of 1 μg/kg

*denotes *p* value < 0.05 and ns denotes *p* value ≥ 0.05

**denotes *p* value < 0.01

### Aflatoxin contamination in crop samples

**Fig 2:**
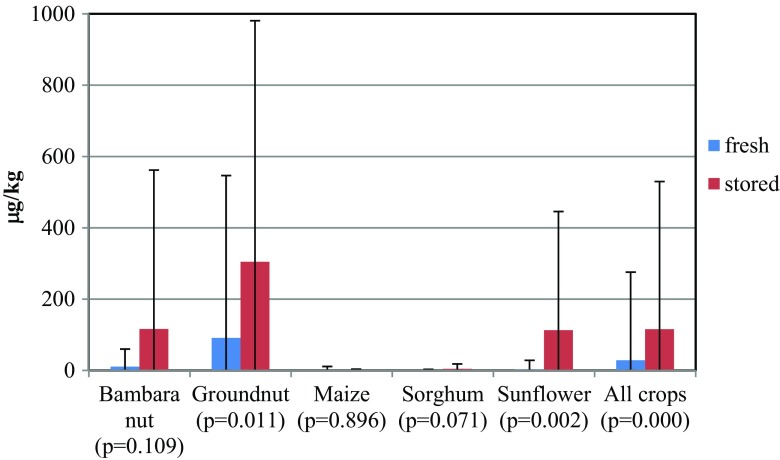
Baseline comparison of AFB_1_ levels between freshly harvested and stored samples in 2012–2013 in central Tanzania. NB: The *p* values are for the two-sample *t* test with unequal variance

**Fig. 3:**
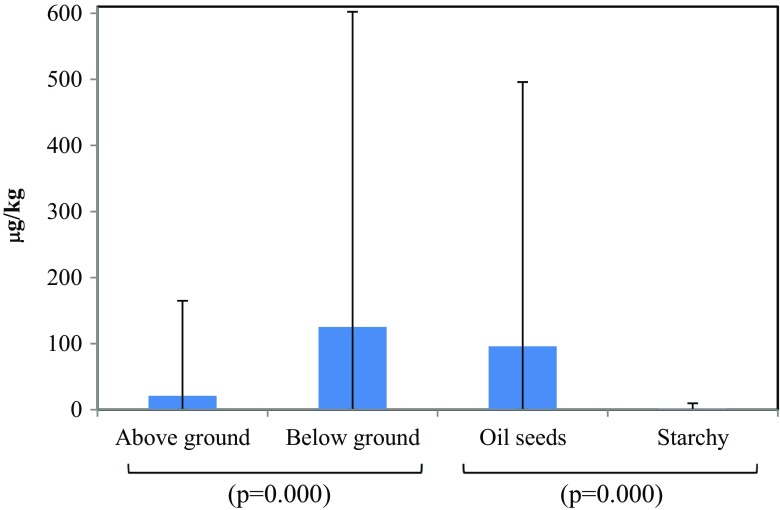
Baseline comparison of AFB_1_ levels between different types of crops produce: kernels/grains produced above ground versus pods produced below ground and oil seed versus starchy crop samples. NB: The *p* values are for the two-sample *t* test with unequal variance

In the cropping season 2012–2013, mean AFB_1_ contamination was 28.7 μg/kg in freshly harvested grains/kernels and 116 μg/kg in stored crop produce (Fig. 2). Mean AFB_1_ contamination in sunflower, maize, and sorghum was 21.0 μg/kg compared to 125 μg/kg in groundnut and bambara nut, which were more exposed to soil during crop grow (Fig. 3). Mean AFB_1_ contamination in oilseeds from sunflower and groundnut was 95.9 μg/kg compared to 1.4 μg/kg in starchy cereals of maize and sorghum (Fig. 3). These differences were highly significant (*p <* 0.000). On average, over the two cropping seasons (2012–2013 and 2013–2014), the villages of Mlali, Moleti, and Njoro showed low AFB_1_ content in freshly harvested grain with relatively higher aflatoxin levels in stored grain/kernels (Table 2). Meeting the maximum recommended levels of AFB_1_ contamination of <10 μg/kg in crop produce was largely influenced by crop type and storage duration (Table 3). Moreover, samples obtained from grain/oilseed markets in the region revealed that nearly all oilseed crops represented by groundnuts were contaminated with AFB_1_ above the permissible standard compared to 26% of maize samples, a starchy grain.

**Table 3:** AFB_1_ content in freshly harvested and stored grain samples over two cropping seasons of 2012–2013 and 2013–2014 in central Tanzania

Year/cropping season	Crop	Frequency of contamination			
		Freshly harvested grain/kernels		Stored grain/kernels	
		Number of samples	Samples > 10 (μg/kg) of aflatoxin (%)	Number of samples	Samples > 10 (μg/kg) of aflatoxin (%)
2012–2013	Bambara nut	78	6.4	48	62.5
	Groundnut	163	18.4	83	81.9
	Sunflower	138	2.1	96	61.4
	Sorghum	57	0.0	40	10.0
	Maize	366	1.9	96	0.0
2013–2014	Bambara nut	64	1.5	131	1.5
	Groundnut	112	5.3	137	6.5
	Sunflower	131	9.1	183	3.8
	Sorghum	35	8.5	137	18.2
	Maize	166	3.6	235	0.9

The results of ordinary least squares regression are presented in Table 4, showing positive and significant coefficients of the groundnut-bambara nut dummy and storage dummy. The AFB_1_ concentration decreases by 38.6 μg/kg on an average if groundnut and bambara nut are not directly exposed to *A. flavus*. Similarly, the marginal effect of storage vis-à-vis fresh samples was +40.2 μg/kg. Remarkably, the effect of being an oilseed crop on AFB_1_ concentration was not statistically significant (*p* = 0.952) after controlling for other covariate variables.

**Table 4:** Quantification of the effects various factors on AFB_1_ contamination using ordinary least squares (OLS) multiple regression estimates, 2013–2014

Dependent variable = AFB_1_ concentration in crop sample (μg/kg)			
Explanatory variable	Coefficients	Standard error	*p* value
Groundnut-bambara nut dummy (1 if groundnut or bambara nut)	38.614	12.294	0.002
Oilseeds dummy (1 if oilseed)	0.713	11.752	0.952
Storage dummy (1 if stored)	40.172	9.197	0.000
2014 dummy (1 if 2014)	−48.809	9.581	0.000
Intercept	69.693	15.766	0.000

Number of observations = 2485; *R*-squared = 0.0231; *F*-statistic (4. 2480) = 14.67 (*p* < 0.000)

### Farmer learning and adoption of aflatoxin mitigation innovations

**Fig. 4:**
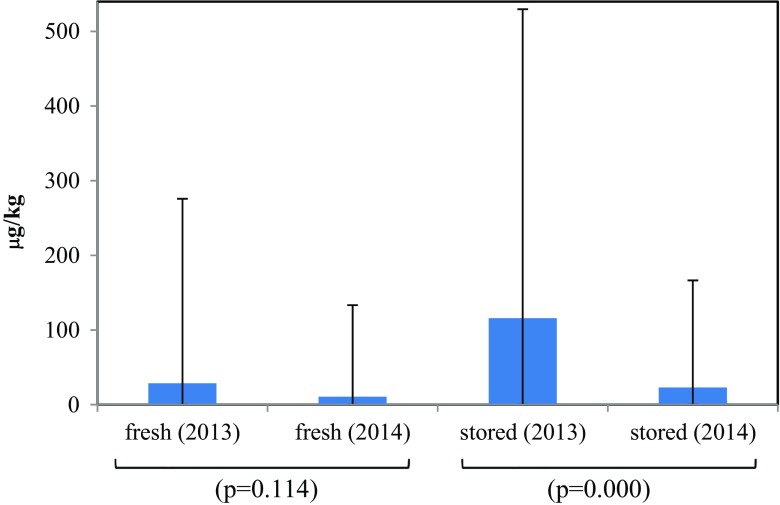
Comparison of AFB_1_ levels in fresh and stored grain/kernels samples, central Tanzania. NB: The *p* values are for the two-sample *t* test with unequal variance

Tracking studies involving 188 farmers of the 312 farmers engaged in the study, revealed a major increase from 19 to 82% of farmers with knowledge regarding the negative outcomes of AFB_1_ on health. Indeed, 82% of farmers who had previously dried their groundnut on the ground shifted to drying them in the field using the Mandela Cork ventilated system or by stacking groundnut stalks with pods exposed to the sun for 3 weeks in the field. Following education, for maize and sorghum, farmers dried cobs and ears to the proper moisture content before storage on polythene sheets rather than on bare earth, limiting exposure to *Aspergillus* and other fungal infections. Adoption of grading to remove unhealthy and rotten grains/kernels increased remarkably from 14 to 78% (Table 1). A dramatic decrease in stored grains/kernels with AFB_1_ levels above the approved levels was detected among samples subjected to the recommended practices. The contamination frequency of 44.4% in the 2012–2013 baseline period decreased to 5.9% in 2014. The mean AFB_1_ contamination level in the stored samples also decreased from 116 μg/kg in 2012–2013 to 23.0 μg/kg in 2013–2014 (Fig. 4).

Regression analysis revealed a positive and significant effect of farmer training and learning. If non-trained farmers receive the training provided in this study, aflatoxin concentration is predicted to be decreased by 28.9 μg/kg on average.

## Discussion

### Predisposition to aflatoxin contamination

In the current study, aflatoxigenic *A*. *flavus* was present in soil samples from all villages. However, the frequency of aflatoxigenic *Aspergillus* species in soil was not associated with AFB_1_ contamination in crop products, particularly in groundnut (Table 2). Predisposition of crops to AFB_1_ contamination was influenced by time of planting, crop variety, post-harvest handling, and storage conditions (Diao et al. 2015). However, regardless of the year and crop type, freshly harvested grain/kernels had significantly lower levels of AFB_1_ compared to stored grain/kernels. These findings demonstrate that *A*. *flavus* infection begins in the field and increases during storage because of inappropriate post-harvest handling methods used by farmers and other value chain actors. For instance, drying crop produce on the ground supports fungal contamination allowing colonization and production of AFB_1_. Interestingly, the level of AFB_1_ contamination in maize was not significantly different between freshly harvested and stored crop products, suggesting a limited increase in contamination of grain from the field to storage. However, other mycotoxins associated with fungal infection of maize have been reported in Malawi and Tanzania (Matumba et al. 2015; Kimanya et al. 2008). The regular consumption of maize as thick maize porridge known as *Ugali* in most Eastern and Southern African countries compared to groundnuts, further increase the risk of chronic exposure to AFB_1_. However, groundnut, a crop highly susceptible to *A*. *flavus* infection that is mainly used as a condiment in child weaning foods or as complete food, also poses a relatively high risk.

The difference in AFB_1_ concentration levels between oilseed crops and starchy crops, suggested by bivariate analysis, was not statistically significant as revealed by multiple regression analysis. This is presumably because the spatial position of grain/kernel production, and its direct exposure to soil fungus is a confounding factor that affects AFB_1_ concentration levels, while being correlated by accident with grain/kernel type (i.e., oilseed or starchy grain).

### Mitigation efforts and farmer learning

Our study confirms that smallholder farmers in central Tanzania use inappropriate post-harvest handling practices, which aggravates AFB_1_ contamination of their crop produce. To improve food safety, it is imperative that farmers be made aware of the hazards of AFB_1_ and that mitigation technologies be promoted. This study showed that interventions produced a six-fold increase in awareness of appropriate post-harvest handling methods for AFB_1_ mitigation. Moreover, farmers were able to see the difference in terms of quality of grain/kernel after using post-harvest crop handling methods. Farmers who adopted mitigation options had over 80% of their crop produce having the acceptable levels of AFB_1_ contamination. These results show that increased knowledge regarding risks associated with AFB_1_ contamination and training on mitigation technologies increased adoption of mitigation innovations. Considering the high AFB_1_ contamination level in groundnut and bambara nut, compared to other crops in this mixed cropping system, it is important to deploy integrated aflatoxin mitigation methods. This requires deployment of bio-control options and post-harvest crop management practice to minimize colonization and subsequent contamination of grain/kernels by AFB_1_.

## References

[CR1] Abbas HK, Shier WT, Horn BW, Weaver MA (2004). Cultural methods for aflatoxin detection. J Toxicol Toxin Rev.

[CR2] Bosch FX, Ribes J, Diaz M, Cleries R (2004). Primary liver cancer: worldwide incidence and trends. Gastroenterol.

[CR3] Cressie NAC, Whitford HJ (1986). How to use the two sample t-test. Biom J.

[CR4] Diao E, Dong H, Hou H, Zhang Z, Ji Z, Ma W (2015). Factors influencing aflatoxin contamination in before and after harvest peanuts: a review. J Food Res.

[CR5] Dismuke C, Lindrooth R (2006) Ordinary least squares. Methods and Designs for Outcomes Research 93

[CR6] Gong YY, Egal S, Hounsa A, Turner PC, Hall AJ, Cardwell KF, Wild CP (2003). Determinants of aflatoxin exposure in young children from Benin and Togo, West Africa: the critical role of weaning. Int J Epidemiol.

[CR7] Guchi E (2015). Aflatoxin contamination in groundnut (*Arachis hypogaea* L.) caused by *Aspergillus* species in Ethiopia. Appl Environ Microbiol.

[CR8] Hall AJ, Wild CP, Eaton DA, Groopman JD (1994). Epidemiology of aflatoxin related disease. The toxicology of aflatoxins: Human health, veterinary and agricultural significance.

[CR9] Hell K, Cardwell KF, Setamoub M, Poehling HM (2000). The influence of storage practices on aflatoxin contamination in maize in four agroecological zones of Benin, west Africa. J Stored Prod Res.

[CR10] Horn BW, Greene RL, Dorner JW (1995). Effect of corn and peanut cultivation on soil populations of *Aspergillus flavus* and *A. parasiticus* in southwestern Georgia. Appl Environ Microbiol.

[CR11] International Crops Research Institute for the Semi-Arid Tropics (ICRISAT) (2012) Collective action and reaction: Market-based groundnut development in Malawi. ICRISAT Eastern and Southern Africa 2011 Highlights, 17–21. (oar.icrisat.org/6659/1/Collective_action_and_reaction.pdf). Accessed 29 Sept 2016

[CR12] Jaime-Gracia R, Cotty PJ (2006). Spatial relationship of soil texture and crop rotation to *Aspergillus flavus* community structure in South Texas. Ecol Epidemiol.

[CR13] Jolly PE, Inusah S, Lu B, Ellis WO, Nyarko A, Phillips TD, Williams JH (2013). Association between high aflatoxin B_1_ levels and high viral load in HIV-positive people. WMJ.

[CR14] Kimanya ME, De Meulenaer B, Tiisekwa B, Ndomondo-Sigonda M, Devlieghere F, Van Camp J (2008). Co-occurrence of fumonisins with aflatoxins in home-stored maize for human consumption in rural villages of Tanzania. Food Addit Contam Part A: Chem Anal Control Expo Risk Assess.

[CR15] Klich MA (2007). *Aspergillus flavus*: the major producer of aflatoxin. Mol Plant Pathol.

[CR16] Klich MA, Pitt JI (1988). Differentiation of *Aspergillus flavus* from *Aspergillus parasiticus* and other closely related species. Trans Br Mycol Soc.

[CR17] Lin MT, Dianese JC (1976). A coconut-agar medium for rapid detection of aflatoxin production by *Aspergillus* spp. Phytopathology.

[CR18] Mahuku G, Henry SN, Waliyar F, Diarra B, Kodio O (2010) Aflatoxin prevalence and data collection. Sampling framework and methodology. Working paper 1. Aflacontrol 1–17

[CR19] Matumba L, Van Poucke C, Ediage EN, Jacobs B, De Saeger S (2015). Effectiveness of hand sorting, flotation/washing, dehulling and combinations thereof on the decontamination of mycotoxin-contaminated white maize. Food Addit Contam.

[CR20] Mehl HL, Cotty PJ (2013). Influence of plant host species on intraspecific competition during infection by *Aspergillus flavus*. Plant Pathol.

[CR21] Monyo ES, Njoroge SMC, Coe R, Osiru M, Madinda F, Waliyar F, Thakur RP, Chilinjika T, Anitha S (2012). Occurence and distribution of aflatoxin contamination in groundnuts (*Arachis hypogaea* L) and population density of *Aflatoxigenic Aspergilli* in Malawi. Crop Prot.

[CR22] Otsuki T, Wilson JS, Sewadeh M (2001). What price precaution? European harmonization of aflatoxin regulations and African groundnut exports. Euro Rev Agri Econ.

[CR23] Park DL, Trucksess MW, Nesheim S, Stack M, Newell RF (1994). Solvent-efficient thin-layer chromatographic method for the determination of aflatoxins B1, B2, G1, and G2 in corn and peanut products: collaborative study. J AOAC Int.

[CR24] R Development Core Team (2014) R: A language and environment for statistical computing. The R Foundation for Statistical Computing, Vienna, Austria. URL http://www.R-project.org/

[CR25] Reddy SV, Kiran Mayi D, Uma Reddy M, Thirumala-Devi K, Reddy DVR (2001). Aflatoxins B1 in different grades of chillies (Capsicum annum L.) in India as determined by indirect competitive ELISA. Food Addit Contam.

[CR26] SAS Institute Inc. (2013) SAS/STAT® 12.3 User’s Guide, Cary

[CR27] Shirima CP, Kimanya ME, Routledge MN, Srey C, Kinabo JL, Humpf HU, Wild CP, Tu YK, Gong YY (2015). A prospective study of growth and biomarkers of exposure to aflatoxin and fumonisin during early childhood in Tanzania. Environ Health Perspect.

[CR28] Sudini H, Srilakshmi P, Vijay Krishna Kumar K, Njoroge SMC, Osiru M, Seetha A, Waliyar F (2015). Detection of aflatoxigenic *Aspergillus* strains by cultural and molecular methods: a critical review. Afr J Microbiol Res.

[CR29] TFNC (2014) Tanzania Food and Nutrition Centre. Tanzania National Nutrition Survey, 2014, Final Report. The United Republic of Tanzania Ministry of Health and Social Welfare

[CR30] Tsusaka TW, Msere HW, Gondwe L, Madzonga O, Clarke S, Siambi M, Okori P (2016). Assessing the post-harvest constraints in smallholders’ groundnut production: a survey in central Malawi. Agric Sci Res J.

[CR31] Turner PC, Sylla A, Gong YY, Diallo MS, Sutcliffe AE, Hall AJ, Wild CP (2005). Reduction in exposure to carcinogenic aflatoxins by postharvest intervention measures in west Africa: a community-based intervention study. Lancet.

[CR32] Wagacha JM, Muthomi JW (2008). Mycotoxin problem in Africa: current status, implications to food safety and health and possible management strategies. Int J Food Microbiol.

[CR33] WHO (2006) World Health Organization. Impacts of aflatoxins on health and nutrition. A report of an expert group meeting Brazzaville, 24–27 2005. World Health Organization regional office for Africa, Brazzaville

[CR34] Wild CP (2007). Aflatoxin exposure in developing countries: the critical interface of agriculture and health. Food Nutr Bull.

[CR35] Williams JH, Phillips TD, Jolly PE, Stiles JK, Jolly CM, Aggarwal D (2004). Human aflatoxicosis in developing countries: a review of toxicology, exposure, potential health consequences, and interventions. Am J Clin Nutr.

[CR36] Wu F, Khlangwiseta P (2010). Health economic impacts and cost-effectiveness of aflatoxin-reduction strategies in Africa: case studies in biocontrol and post-harvest interventions. Food Addit Contam Part A.

[CR37] Yu MC, Yuan JM (2004). Environmental factors and risk for hepatocellular carcinoma. Gastroenterol.

